# Transcatheter and intraoperative device closure of atrial septal defect in infants under three years of age

**DOI:** 10.1186/s13019-020-1063-z

**Published:** 2020-01-08

**Authors:** Yangyang Han, Xiquan Zhang, Fengwei Zhang

**Affiliations:** 1 Department of Cardiovascular Surgery, Linyi People’s Hospital, Affiliated Hospital of Shandong University, Jiefang Street No. 27, Linyi, 276000 Shandong Province China; 2 Department of Cardiovascular Surgery, Qilu Hospital of Shandong University, West wenhua road No.107, Lixia district, Jinan, 250012 Shandong province China

**Keywords:** Atrial septal defect, Intraoperative device closure, Percutaneous closure, Occluder, Infant

## Abstract

**Background:**

Transcatheter and intraoperative device closures have been widely used in the treatment of secundum atrial septal defect (ASD). However, for young infants with ASD, device closure remains controversial, and such treatment features limited data. We compared the clinical data and follow-up results of percutaneous and intraoperative device closure for ASD to evaluate the feasibility, safety, and efficacy of both treatments in infants under 3 years of age.

**Methods:**

From September 2010 to September 2018, 186 children under 3 years of age with significant secundum ASD were included in this study. A total of 88 and 98 patients were divided into groups A (transcatheter device closure) and B (intraoperative device closure), respectively. The clinical data and follow-up results of the two groups were analyzed retrospectively.

**Result:**

The mean age and weight of patients in group A were significantly higher than those in group B. The proportion of complex ASDs (multiples or rims deficiency) and the device/weight ratio in group B were significantly higher than those in group A. Successful closure was obtained in 86 (97.7%) and 96 (98.0%) infants in groups A and B, respectively, with two failed cases in each group (2.3% vs 2%). The rate of periprocedural complications reached 13.6 and 26.5% for groups A and B (*P* = 0.058), respectively. The durations of the procedure and postoperative hospital stay in group A were significantly shorter than those in group B (*P* < 0.05). Excellent follow-up results were observed in both groups. At present, no death nor major complications have occurred. Symptoms either resolved completely or improved significantly for all symptomatic infants. No residual shunts at the 6th month of follow-up evaluation were observed. Patients with failure to thrive gained weight appropriately for age, and the structure and hemodynamic parameters significantly improved during follow-up.

**Conclusion:**

Transcatheter and intraoperative device closure are feasible, effective, and safe methods for the treatment of ASDs in infants under 3 years of age. Considering improved cosmetic effect and the short duration of the procedure and postoperative hospital stay, transcatheter is preferred for patients with appropriate conditions. Intraoperative device closure can be performed as an alternative to percutaneous closure, particularly for infants with large, complex ASDs, young age, or low-body weight.

## Background

Atrial septal defect (ASD) is a common cardiac malformation that accounts for approximately 7–10% of congenital heart diseases [1]. ASD in infants is usually asymptomatic, and despite the increase in right cardiac load, the patients can usually wait until they are over 3 years old or weighing more than 15 kg before undergoing closure without affecting their growth and development. However, the closure of ASD at a young age may be beneficial, especially for preterm infants [2–4]. The closure of ASD must be performed in advance for young patients with congestive heart failure, frequent respiratory infection, failure to thrive, or significantly elevated pulmonary arterial pressure [5, 6]. Traditional surgical closure that requires extracorporeal circulation is safe, effective, and feasible for nearly all patients with ASD [7]. However, the traditional method exhibits disadvantages, such as large trauma, numerous complications, and long postoperative recovery time and surgical incision, that may cause substantial psychological trauma to the growth of children. After years of development and improvement, fluoroscopy and ultrasound-guided transcatheter closure technique have gradually become another standard treatment for most secundum ASDs [8]. However, the use of transcatheter closure presents difficulty and limitations in small children and infants with large defects [9]. In recent years, minimally invasive transesophageal echocardiography (TEE)-guided transthoracic occlusion has also been developed and applied in China. This technique can be used to directly puncture into the right atrium through a minimally invasive thoracic incision to avoid the possible damage of fluoroscopy and contrast agents to the body and the possibility of peripheral vascular injury [10–15]. For infants for whom percutaneous occlusion is unsuitable, transthoracic minimally invasive occlusion may be a good alternative. The comparison of these methods in the treatment of ASDs in infants (less than 36 months old) has never been reported.

In this work, we report our single-center experience in the comparison of procedural and follow-up results of transcatheter versus intraoperative device closure for ASDs in infants. This study aimed to explore the safety, feasibility, and effectiveness of these techniques and to provide a good treatment strategy for ASD in infants.

## Methods

### Patients

This study was approved by the Ethics Committee of Linyi People’s Hospital Affiliated to Shandong University. An informed written consent was obtained from each patient for every procedure.

From September 2010 to September 2018, 186 children under 3 years of age with significant secundum ASD underwent ASD device closure. The patients were divided into two groups in accordance with the closure approach. A total of 88 and 98 patients were included in groups A (transcatheter device closure) and B (intraoperative device closure), respectively. The indication for ASD closure was hemodynamically significant left-to-right shunt (pulmonary to systemic flow ratio > 1.5 measured by echocardiography), which was manifested by the enlargement of the right ventricle (RV). Other indications included failure to thrive, frequent respiratory infections, increased sweating and easy fatigue, significantly elevated pulmonary arterial pressure, or strong parental request. All children were diagnosed with secundum ASD without the presence of other cardiac malformations, elevated nonreactive pulmonary vascular resistance, uncontrolled congestive heart failure, and any infection that could not be successfully treated before device closure. In general, the inclusion criteria for group A were as follows: secundum ASD with the presence of adequate rims (≥5 mm); the distance from the defect edge to the coronary sinus, superior and inferior vena cava, and pulmonary vein was ≥5 mm, and that until the atrioventricular flap reached 7 mm; suitable body weight(≥10 kg) with good peripheral vascular development. The inclusion criteria for group B were as follows: inclusion criteria for group A; no specific requirement for patient weight; secundum ASD with deficient rim < 1/4. Before treatment selection, all the guardians of the patients were informed of the indications, advantages and disadvantages, and specific risks of both treatments. For example, transcatheter device closure involves a short postoperative recovery time and zero incision. The intraoperative device closure requires minimal incision but no vascular injury and X-ray exposure and can be immediately converted to surgical repair in the operating room. In general percutaneous closure is preferred for patients who satisfied the inclusion criteria for group A, whereas the final treatment plan must be considered along with the preferences of the patient’s guardian. Table 1 summarizes the patients’ clinical data regarding sex, age, body weight, ASD sizes, indications, and characteristics. Routine clinical examinations before the procedure included transthoracic echocardiography (TTE), chest X-rays, electrocardiogram, and blood and biochemical tests. The ASD diameter and atrial septum length were measured in several views by echocardiography, and the largest measurement was recorded as the ASD diameter. Pulmonary pressures were assessed by the tricuspid and pulmonary regurgitations and ventricular septal shape.

**Table 1:** Comparison of baseline characteristics of both groups

Characteristics	Group A	Group B	P
Number of patients	88	98	
Male/female	38/50	45/53	0.708
Age (months)	28.3 ± 6.6 (11–36)	16.4 ± 8.0 (5–36)	< 0.05
Weight (kg)	12.9 ± 1.5 (9.5–16)	9.7 ± 1.8 (5.9–14)	< 0.05
ASD size (mm)	9.1 ± 2.6 mm	9.6 ± 2.7 mm	0.233
Indications			
RV dilatation	88 (100%)	98 (100%)	NS
Failure to thrive	28 (31.8%)	39 (39.8%)	0.286
Frequent respiratory infections	21 (23.9%)	32 (32.7%)	0.185
Easy fatigue	16 (18.2%)	17 (17.3%)	0.882
Pulmonary hypertension	5 (5.7%)	10 (10.2%)	0.258
Strong parental request	18 (20.5%)	0 (0%)	< 0.05
Multiples	2 (2.3%)	15 (15.3%)	< 0.05
Deficient rims	3 (3.4%)	17 (17.3%)	< 0.05
Posterior rim	1	5	
Aortic and superior rim	2	8	
Inferior rim	0	4	
Down syndrome	3 (3.4%)	5 (5.1%)	0.57
Atrial septal aneurysm	0	6 (6.1%)	< 0.05

*A:* transcatheter device closure, *B:* intraoperative device closure, *ASD:* atrial septal defect, *NS:* not significant. Continuous data are presented as mean ± standard deviation, and categorial variables are presented as number and percentage

### Device

A domestic ASD device (Shanghai Shape Memory Alloy Co., Ltd., Shanghai, China) made from nickel and titanium alloys was used in both groups. The ASD occluder was modified from the Amplatzer atrial septal occluder. A standard transfemoral approach was adopted in group A. The delivery system, which consisted of a sheath and a pushing rod, used in group B was shorter than that in group A, which was specially designed for intraoperative device closure.

### Procedure

General anesthesia with endotracheal intubation and antibiotic prophylaxis were routinely given to all patients before the procedure. Endotracheal intubation was removed in the operating or recovery room after the procedure. Patients were transferred to the general ward after their condition stabilized.

In group A, heparin (100 IU/kg) was given to all patients before femoral venous puncture. All procedures were performed using TTE/TEE monitoring and X-ray guidance in cardiac catheterization room. The size and morphology of defects were evaluated by TTE/TEE. The procedure was usually performed in accordance with the standard protocol, whereas several cases of pulmonary vein deployment technique could be used to facilitate the transcatheter closure of large secundum ASDs.

The intraoperative device closure technique used in group B has been described in previous studies [10–15]. In brief, the TEE probe was inserted after general anesthesia with endotracheal intubation was given to patients, and ASD closure was performed by only using TEE guidance. A minimally invasive incision (2 cm to 3 cm) was made in the right anterior third or fourth intercostal space of the right sternal border. Then, a mini-retractor was used in this manipulation incision. The pericardium was opened and cradled to suspend the heart. A 5–0 prolene purse–string suture was stitched on the right atrium. A secure suture of 3–0 prolene was passed through the selected device under the microscrew. Then, the selected device was loaded into a delivery sheath. Heparin (100 IU/kg) was administered to all patients before the loaded sheath was inserted into the purse-string suture of the right atrium. The loaded sheath was passed through the ASD into the left atrium under TEE guidance. The left umbrella folder, waist, and the right umbrella folder were then deployed to close the ASD. After the device was successfully implanted, suitable views were used to assess the position and possible impacts of the occluder on surrounding structures. Color doppler flow was used to observe atrioventricular valve function, coronary sinus return, systemic/pulmonary venous return, and residual shunting. After the occluders were implanted in place, they were released while keeping the secure suture connected with the device for 3–5 min to observe the device position. The secure suture was then removed with the purse-string tied. The minimally invasive incision was closed without a drainage tube. Before the incision was closed, the aspirator was used to completely remove the fluid in the right chest cavity and pericardium and empty the air by expanding the lungs.

### Follow-up

The day following the procedure, TTE, chest X-rays, and electrocardiogram were performed to check for device position, any residual shunt and arrhythmia, or other peri-procedural complications. All patients were discharged with aspirin medication (3–5 mg/kg body weight, orally) for 6 months. At 1, 3, 6, and 12 months, all patients underwent another TTE and electrocardiogram. Further annual clinical follow-up was scheduled for all patients. The main concern was whether translocation and thrombosis formation of the occluder, new arrhythmia, any residual shunt, aortic erosion, and other complications occurred.

### Statistical analysis

Statistical analysis was performed using the SPSS 17.0 software (SPSS Inc., Chicago, IL). Continuous data are presented as mean ± standard deviation, and categorial variables are presented as number and percentage. Differences between the groups were analyzed using independent-samples t-test for continuous variables and χ2 test for categorical variables. *P* < 0.05 was considered significant.

## Results

### Baseline characteristics

As summarized in Table 1, statistically significant differences were observed in certain demographic data or preoperative clinical characteristics between the two groups. The mean age of patients was 28.3 ± 6.6 and 16.4 ± 8.0 months for groups A and B (*P* < 0.05), respectively. In group B, the mean body weight was significantly lighter than that in group A (9.7 ± 1.8 kg versus 12.9 ± 1.5 kg, *P* < 0.05). The proportion of complex ASDs (multiples or rims deficiency) in group B was significantly higher than that in group A.

### Intraoperative and post-operative results

Table 2 lists the intraoperative and post-operative results. Successful closure was obtained in 86 (97.7%) and 96 (98.0%) infants in groups A and B, respectively, with two failed cases in each group (2.3% vs 2%). An immediate device embolization in the RV occurred in group A, and the patients were referred to emergency surgery for device retrieval and surgical ASD closure. During rescue, device embolization caused cardiac arrest and blood flow interruption, causing minor brain complication that gradually improved after treatment. Percutaneous device closure was aborted in another child due to the oblique position of the implant in a relatively large defect. Intraoperative closure was successfully performed 1 week later in this child. In group A, the initially unstable implanted device was replaced by a second larger device in three patients. In group B, intraoperative device closure was abandoned in two patients because the devices were difficult to fix for the deficient inferior rims of ASDs. We immediately extended the incision to replace the device closure with a right chest surgical closure. In group A, the diameter of ASDs, as measured by TTE/TEE, ranged from 5 mm to 16 mm (9.1 ± 2.6 mm), whereas the size of the implanted occluder ranged from 8 mm to 22 mm (12.6 ± 3.5 mm). In group B, the corresponding data were 6 mm to 20 mm diameter (9.6 ± 2.7 mm, *P* = 0.233) and 8 mm to 24 mm device size (13.0 ± 3.0 mm, *P* = 0.404). In all patients, the ratio of the applied device size to body weight was determined. The device/weight reached 0.97 ± 0.23 and 1.35 ± 0.27 in groups A and B (*P* < 0.05), respectively, and the proportion of using a considerably large occluder (device/weight ratio ≥ 1.5) was significantly lower in group A than that in group B. Trivial residual shunts were documented immediately after the procedure in 3 and 8 patients in groups A and B (3.4% vs 8.2%, *P* = 0.17), respectively. The durations of the procedure and the postoperative hospital stay in group A were significantly shorter than those in group B (*P* < 0.05). Mean fluoroscopy time in group A was 4.6 ± 7.9 min.

**Table 2:** Intraoperative and post-operative results on transcatheter and intraoperative device closure

	Group A	Group B	P
Successful closure, *n* (%)	86 (97.7%)	96 (98.0%)	0.913
Device size	12.6 ± 3.5	13.0 ± 3.0	0.404
Device/weight ratio	0.97 ± 0.23	1.35 ± 0.27	< 0.05
Device/weight ratio ≥ 1.5, *n* (%)	3 (3.4%)	28 (28.6%)	< 0.05
Time of procedure (mins)	41.4 ± 22.7	66.3 ± 20.9	< 0.05
Postoperative hospital stay (days)	1.8 ± 1.1	2.6 ± 1.3	< 0.05
Follow-up (months)	52.1 ± 26.5	58.1 ± 28.0	0.134
Residual shunting	3 (3.4%)	8 (8.2%)	0.17
Periprocedural complications	12 (13.6%)	26 (26.5%)	0.058
Cardiac death	0	0	NS
Device embolization	1	0	0.29
Cardiac perforation	0	0	NS
Hydrothorax or Pericardial effusion	0	5	< 0.05
Transient cardiac arrhythmias	8 (9.0%)	17 (17.3%)	0.099
Hematoma at access site	3 (3.4%)	0	0.065
Surgical wound complications	0	2 (2%)	0.178
Pneumonia	0	2 (2%)	0.178
Incision length (cm)	/	2.4 ± 0.9	
Fluoroscopy time (mins)	4.6 ± 7.9	/	

*A:* transcatheter device closure, *B:* intraoperative device closure, *NS:* not significant. Continuous data are presented as mean ± standard deviation, and categorial variables are presented as number and percentage

Death, erosion, tamponade, cardiac perforation, atrioventricular valve distortion, endocarditis, thromboembolism, or permanent rhythm disturbances were absent in both groups. The rate of periprocedural complications totaled 13.6 and 26.5% for groups A and B (*P* = 0.058), respectively. In group A, minor complications, including transient cardiac arrhythmias during the procedure and hematoma at the access site, were encountered in 11 patients (12.5%). Among these complications, first-degree atrioventricular block (AVB) and frequent ventricular premature beats were noted three patients. Atrial fibrillation (AF) was observed in two patients. Hematoma at the access site occurred in three patients without requiring medical intervention. In group B, transient cardiac arrhythmias were documented in 17 patients periprocedurally; these arrhythmias included AF, atrial tachycardia, atrial flutter, frequent ventricular premature beats, and second-degree AVB in 6, 4, 2, 4, and 1 patient, respectively. All the patients were easily treated by medicine or automatic recovery. Pericardial effusion or hydrothorax was observed in five patients. One patient required drainage. Two patients developed pneumonia and received antibiotic treatment. Delayed incision healing occurred in two patients due to the overextension injury of retractor pulling or electrotome burn.

### Follow-up results

**Fig. 1:**
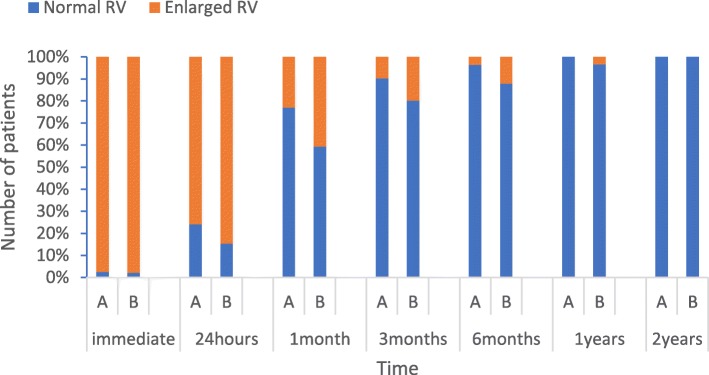
Changes in RV after the device closure of ASD depending on the length of the follow-up period. A: Group with transcatheter device closure; B: group with intraoperative device closure; RV: right ventricle; Enlarged RV: the ratio of right ventricular to left ventricular end-diastolic transverse dimension (RV/LV) ≥ 1; Normal RV: RV/LV < 1

Among the 184 patients who had a successful ASD closure, follow-up data were available for 83 (96.5%) and 91 (94.8%) patients in groups A and B, respectively. Three (3.5%) and five (5.2%) patients in groups A and B were lost to follow-up, respectively. After a median follow-up duration of 46 months (mean: 52.1 ± 26.5 months; range: 14–102 months) in group A and 58 months in group B (mean: 58.1 ± 28.0 months; range: 13–107 months), no death, thromboembolic events, aortic erosions, occluder dislodged, and other major complications were observed. At present, symptoms either resolved completely or improved significantly for all symptomatic infants. One patient in group B had trivial shunts at the 3-month follow-up evaluation, and none exhibited any residual shunts at the 6-month follow-up evaluation. In mid-term to long-term observation, mild mitral valve insufficiency occurred in one patient in group B. Pulmonary hypertension resolved in both groups as estimated by echocardiography, and patients with failure to thrive gained weight appropriately for their age. In both groups, the structure and hemodynamic parameters significantly improved during follow-up. RV end-diastolic anteroposterior dimension and the ratio of right ventricular to left ventricular end-diastolic transverse dimension (RV/LV) were recorded before ASD closure. RV dilation was present in all patients. RV dimension and RV/LV decreased in all patients at the two-year follow-up evaluation (Fig. 1).

## Discussion

According to the present study, transcatheter or intraoperative device closure of ASDs is feasible and effective in infants under 3 years of age. Studies on ASD closure in infants mostly involved the results on transcatheter closure [4, 9, 16]. Transcatheter closure has been the first choice in the treatment of secundum ASD with appropriate morphology [8]. However, performing this technique to young children with large ASD or low body weight remains difficult. Small children under percutaneous closure with large ASD have shown a relatively low procedural success rate and significantly high rates of periprocedural and delayed complications [9, 17].

In the present study, the age and weight of group A were significantly higher than those of group B. Furthermore, the proportion of complex ASDs and the use of an extremely large occluder (device/weight ratio ≥ 1.5) in group A were significantly lower than those in group B. An important reason was the relatively demanding inclusion criteria of percutaneous closure for infants. Based on our experience, percutaneous closure is difficult to implement in infants with thin peripheral veins. In addition, considerably large defects are also unsuitable for percutaneous closure because the loading sheath is angulate to the defects in percutaneous closure but vertical to those in intraoperative device closure. “Unbutton effect” may occur in percutaneous closure with the same size of occluder [14]. Although auxiliary technology (pulmonary vein deployment technique or balloon-assisted technique) could be used to facilitate the transcatheter closure of large secundum ASDs, the procedure would inevitably increase the fluoroscopy time and dosage of contrast agent. Radiation can induce cancer and affect the function of organs, such as bone marrow, genitals, and thyroid gland [18–20]. Although radiation-induced cancer with a median follow-up duration of 4.5 years has not been shown in the present study, the effect on infants in the coming decades remains unclear. Thus, minimizing or avoiding the effect of X-ray radiation is necessary. Fidelio, et al. determined that the requirement for balloon calibration and large ASD/device is a part of the risk factors associated with increased radiation dose area product. A low dose of radiation can be achieved for transcatheter ASD closure by avoiding unnecessary maneuvers and using echocardiographic guidance as much as possible [21]. Measuring the ASD diameter in group A with the use of echocardiography instead of balloon could reduce fluoroscopy time and avoid complications associated with balloon measurement.

A high overall incidence of perioperative complications (20.4%) was observed in the two groups, but most of them were minor complications that could be well managed without leaving sequelae. The incidence of early complications showed no significant difference in both groups (13.6% versus 26.5%), but the types of complications varied slightly. Arrhythmia and occlusion failure rates were similar in both groups, the incidence of peripheral vascular injury hematoma was relatively high due to the need of peripheral vascular approach in group A, whereas the incidence of hydrothorax or pericardial effusion was relatively high due to a small incision in the chest in group B. Based on our experienced, only a periodic review was needed for a small amount of hydrothorax or pericardial effusion after procedure. The effusion can be absorbed and eliminated within 1 month. For values higher than the moderate volume of effusion, ultrasonic positioning puncture and catheter drainage were generally required.

Excellent follow-up results were observed in both groups. Existing analysis has shown that residual shunt after device closure of ASD usually resolved spontaneously in the early postoperative period [17]. Our results confirmed this finding, and the complete closure rate of both groups was 100% at 6 months of follow-up. The size of the right heart significantly reduced, the exercise endurance improved, and the incidence of respiratory tract infection significantly decreased in the two groups.

In the present study, an immediate device embolization occurred in group A and caused a minor brain complication. All procedures of group A were performed in cardiac catheterization room. Once severe complications, such as the detachment of occluder and pericardial tamponade, occurred, emergency surgery was required for retrieval or hemostasis. The patient must be transported from the catheter room to the surgical operating room or hybridization room. Re-sterilization and transfer would cause a delay in time and increase risk. However, all procedures of group B can be immediately converted to thoracotomy in the operating room, avoiding a delay in transfer or re-sterilization of the patients during severe complications due to operational failure. In addition, the application of secure suture passing through the selected device under the microscrew can be used to retrieve a suboptimally placed device through a large delivery sheath to avoid device embolization and improve the security of this technique. In the future, the transcatheter device closure of ASDs in infants in our center can be preferably completed in one stop in hybrid operating rooms. Transcatheter device closure can be converted to intraoperative device closure or surgical closure flexibly to maximize the benefits of patients through the coordination of pediatric cardiologists and cardiac surgeons.

### Study limitations

This study was a single-center research performed on selected cases. This work was based on a relatively small series of patients, and the follow-up period was 55.3 ± 27.3 months. A multicenter, prospective, randomized trial is convictive. In addition, 4.3% of all patients were lost to follow-up. Further studies are required to establish additional long-term results in a large patient population.

## Conclusion

Transcatheter and intraoperative device closure are feasible, effective, and safe methods for the treatment of ASDs in infants under 3 years of age. Considering better cosmetic effect and shorter duration of the procedure and postoperative hospital stay, transcatheter is preferred for patients with appropriate conditions. Intraoperative device closure can be performed as an alternative to percutaneous closure, particularly for infants with large, complex ASDs, young age, or low body weight.

## Data Availability

Please contact author for data requests.

## Abbreviations

AF: Atrial fibrillation
ASD: Atrial septal defect
AVB: Atrioventricular block
RV: Right ventricle
RV/LV: The ratio of right ventricular to left ventricular end-diastolic transverse dimension
TEE: Transesophageal echocardiography
TTE: Transthoracic echocardiography
